# P-1839. Elucidating Key Interactions Between Macrophages and the Fungal Pathogen *Coccidioides*

**DOI:** 10.1093/ofid/ofae631.2002

**Published:** 2025-01-29

**Authors:** Jane Symington, Mark Voorhies, Bevin English, Anita Sil

**Affiliations:** University of California San Francisco, San Francisco, California; UC San Francisco, San Francisco, California; University of California San Francisco, San Francisco, California; UC San Francisco, San Francisco, California

## Abstract

**Background:**

In the environment, the fungal pathogen *Coccidioides* grows as hyphae, yet when its spores (arthroconidia) are inhaled by a host, they develop instead into a parasitic form called the spherule. Spherules can be elicited *in vitro* in the presence of specific media, elevated temperatures, and high CO2, yet what triggers this transition *in vivo* and the role of host immune cells in this transition remains largely unknown.

**Methods:**

We performed live imaging of *Coccidioides posadasii* Silveira arthroconidia in the presence or absence of murine bone marrow derived macrophages isolated from wildtype (C57BL/6) mice. Images were taken hourly over a 3-day time course. We recorded the time at which hyphae first appeared, the number of spherules at the final time point, and average size of spherules at the final time point. We utilize fluorescent dyes to determine if arthroconidia are phagocytosed and to assess host cell death. RNAseq was performed on RNA isolated from arthroconidia cultured with macrophages over 48hrs. Experiments were conducted at 37C and 5% CO2.

**Results:**

We observed that the presence of macrophages strongly promotes the ability of *Coccidioides* arthroconidia to transition to spherules at conditions that would otherwise promote formation of hyphae. In wells with macrophages, arthroconidia produced more spherules, and these spherules were generally larger in size than those produced *in vitro* in the absence of host cells. The presence of macrophages also delayed the time that hyphae first appeared. Using a transwell system, we found that physical contact with macrophages was necessary to promote spherule development and reduce hyphae formation. We discovered that *Coccidioides* arthroconidia are phagocytosed by macrophages and spherules can develop within macrophages. RNAseq of *Coccidioides* arthroconidia cultured with macrophages show enrichment of transcripts associated with spherule growth *in vitro* as well as those previously identified from *Coccidioides*-infected murine or human lungs.

**Conclusion:**

Macrophages strongly promote the ability of *Coccidioides* arthroconidia to transition to the parasitic form of *Coccidioides* in a contact-dependent manner. This work creates a foundation for better understanding initial critical interactions between macrophages and *Coccidioides*.

**Disclosures:**

**All Authors**: No reported disclosures

